# Nanotip-based photoelectron microgun for ultrafast LEED

**DOI:** 10.1063/1.4982947

**Published:** 2017-05-16

**Authors:** Gero Storeck, Simon Vogelgesang, Murat Sivis, Sascha Schäfer, Claus Ropers

**Affiliations:** 4th Physical Institute - Solids and Nanostructures, University of Goettingen, Goettingen, Germany

## Abstract

We present the design and fabrication of a micrometer-scale electron gun for the implementation of ultrafast low-energy electron diffraction from surfaces. A multi-step process involving photolithography and focused-ion-beam nanostructuring is used to assemble and electrically contact the photoelectron gun, which consists of a nanotip photocathode in a Schottky geometry and an einzel lens for beam collimation. We characterize the low-energy electron pulses by a transient electric field effect and achieve pulse durations of 1.3 ps at an electron energy of 80 eV. First diffraction images in a backscattering geometry (at 50 eV electron energy) are shown.

## INTRODUCTION

Ultrafast electron diffraction^1^ and microscopy^2^ are rapidly evolving tools for the study of structural dynamics. In recent years, ultrafast variants of numerous techniques employing electrons as structural and spectroscopic probes were developed, including high-energy electron diffraction,^3,4^ transmission electron microscopy,^5–11^ and electron energy loss spectroscopy.^12,13^ One of the particular benefits of electron beams is the high scattering cross-section facilitating surface-sensitive electron diffraction, for example, in reflection high-energy and low-energy electron diffraction (RHEED and LEED). Ultrafast RHEED was implemented early on in Refs. 14 and 15, and its temporal resolution has reached the few-picosecond to femtosecond domain in the past few years.^16,17^ However, because of its grazing incidence geometry, the real strength of RHEED is its *in-situ* capability to characterize growth during epitaxy, rather than to obtain direct representations of the surface structure and symmetry. Some drawbacks of RHEED are enhanced volume contributions for stepped and imperfect surfaces and its restriction to map a limited angular fraction of reciprocal space. Ultrafast low-energy electron diffraction (ULEED), on the other hand, is highly desirable due to LEED's outstanding ability to map atomic-scale surface structures^18^ but has remained particularly challenging experimentally.^19,20^ A main obstacle in the implementation of ULEED lies in achieving ultrashort electron probe pulses at low energies, which are extremely susceptible to pulse spreading in the propagation from the electron source to the sample.^19,21^ Recently, employing nanoscale photocathodes^22–28^ and minimized propagation distances, this limitation was overcome in a compact transmission ULEED setup for the study of structural dynamics in monolayers and ultrathin films.^29^ In a related approach, ultrafast point-projection microscopy was developed^30–32^ and applied in the imaging of charge dynamics.^30^ Extending the ULEED methodology to a backscattering geometry would enable investigations of ultrafast structural processes at surfaces, but, in order to avoid shadowing of the backscattered diffraction pattern, this requires the development of miniaturized photoelectron sources of sufficiently small outer diameters.

**FIG. 1.**
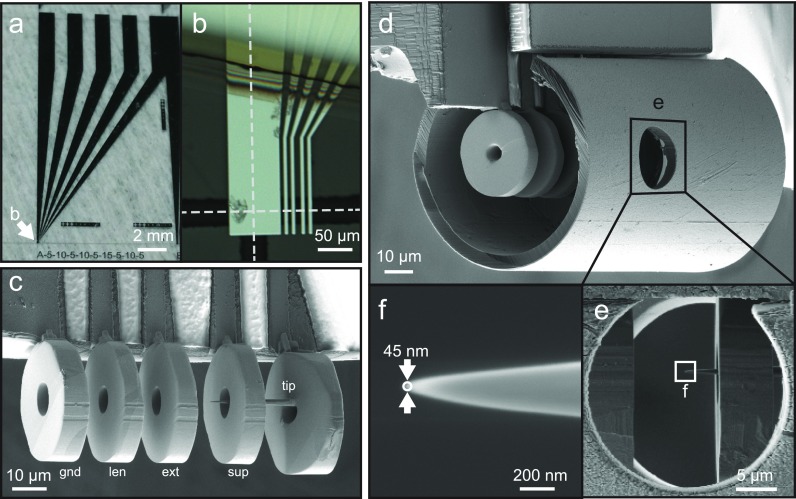
(a), (b) Optical microscope images of photolithographically patterned metallic tracks (Cr on a 70-μm thick Borosilicate glass slide). The electrostatic gun assembly is placed at an edge of the cleaved substrate (dashed lines indicated in (b)). (c)–(f) Scanning electron micrographs of the miniaturized electron gun at various fabrication stages and magnifications. (c) Contacted electrodes of the microgun (without shielding), exhibiting tip, suppressor, extractor, lens, and ground electrodes. (d) Finished microgun with electrostatic shielding attached. (e) Side-view through the laser excitation aperture. (f) Tungsten tip-emitter with a radius of curvature below 50 nm (prepared by focused-ion-beam etching).

Here, we present the implementation of a nanofabricated electron gun (hereafter referred to as the “microgun”) facilitating ULEED. The microgun consists of a tungsten nanotip photoemitter embedded in a shielded micrometer-scale electrostatic lens assembly (total outer diameter of 80 *μ*m; Fig. 1(d)). Utilizing this photoelectron source, we achieve a temporal resolution in electron projection imaging of 1.3 ps at an electron energy of only 80 eV and a source-sample distance of 400 *μ*m. High-quality electron diffraction patterns are recorded in a backscattering geometry, demonstrating the high spatial coherence of the generated electron beam. This photoelectron gun combines ultrafast temporal resolution with high momentum resolution and ultimate surface sensitivity, promoting access to numerous ultrafast phenomena in the structural dynamics at surfaces.

### Gun fabrication

The electrostatic microgun is assembled at the edge of a glass slide, onto which metallic lines (see Figs. 1(a) and 1(b)) are deposited to connect the micrometer-sized gun electrodes to millimeter-scale pads and the voltage supplies. The chromium contact lines are fabricated using a photolithographic process and subsequently covered with an insulating polyimide layer (Kapton). The polymer and the backside of the glass slide are coated with thermally evaporated titanium films to shield electrical stray fields, except for the external contact pads and the strip lines leading to the electron gun (Fig. 1(b)).

The electrode structures comprising the microgun assembly are fabricated by slicing a gold wire using focused-ion-beam etching and are fixed to the edge of a glass slide by ion-beam-induced platinum deposition. In order to minimize the electron propagation distance to the sample, the electrode assembly is placed close to the corner of the contact support. Figure 1(c) displays a scanning electron micrograph of the resulting electron gun with five separate gold electrodes, representing (left to right) the ground, gun lens, extractor, suppressor, and cathode electrodes. The cathode electrode holds a nanometric tungsten tip (focus-ion-beam-prepared, 20 *μ*m tip length, radius of curvature below 50 nm, see Figs. 1(e) and 1(f)). Bright areas in the electron micrograph in Fig. 1(c) are due to electron beam induced charging, indicating sufficient insulation between the contact lines (darker regions). At this stage, the outer diameter of the electron gun is approximately 30 *μ*m. Finally, to minimize electric stray fields, the remaining exposed contact lines and the gun electrodes are shielded by a grounded metal-coated mica plate and a Kapton cylinder, respectively, leading to an effective gun diameter of 80 *μ*m (Fig. 1(d)). The aperture in the shielding hull (indicated with the square in Fig. 1(d)) allows for inducing photoemission from the nanometric tungsten tip by side illumination with laser pulses. The magnified view in Fig. 1(e) shows the tip with its apex located in the center between the suppressor and extractor electrodes (approximately 6 *μ*m from the suppressor electrode).

### Numerical simulations

**FIG. 2.**
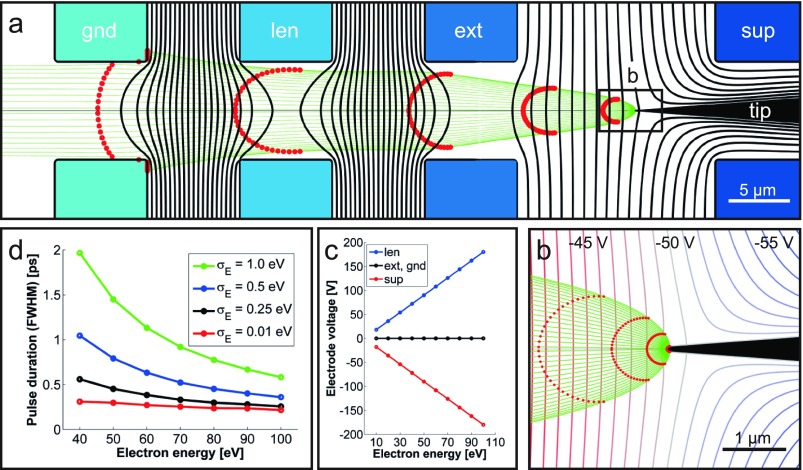
(a) Finite element modeling (FEM) of the miniaturized electron gun for a set of applied electrode voltages described by (tip, sup, ext, len, gnd) = (−50, −90, 0, 90, 0) V. Cylindrical symmetry is assumed. Solid black: equipotential lines, green: electron trajectories. (b) Magnified view of the tip region. (c) Energy-dependent voltage setting applied to electrodes for a collimated electron beam. (d) Pulse duration versus electron energy at a plane 400 *μ*m behind the ground electrode, for various widths in the initial kinetic energy distribution. Red dots in (a) and (b) indicate the positions of groups of electrons emitted at the same time.

In order to estimate the gun performance in terms of temporal resolution and spatial beam parameters, we carried out finite element simulations solving for the electric field and the propagation of electrons in our gun geometry (Fig. 2). Generally, the microgun is composed of a source region including a tip, an extractor and a suppressor electrode, an einzel lens formed by the extractor, a gun lens electrode, and a grounded exit aperture (Fig. 2(a)). Electron trajectories are simulated for a range of voltage settings and initial conditions of the electrons emitted from the hemispherical nanotip apex (green lines in Figs. 2(a) and 2(b)). Assuming one to a few electrons per pulse, we do not consider Coulomb interactions between electrons. For each electron kinetic energy (tip bias plus photoemission excess energy), the suppressor and lens electrode voltages are chosen to form a collimated beam exiting the gun, holding the extractor at ground potential (Fig. 2(c)). Trajectories are obtained for a range of emission positions along the apex (0°–90° from the axis), emission angles (±90° from the surface), and initial kinetic energies (0–3 eV). A total number of about 50.000 particle trajectories are computed for each bias voltage.

From these trajectories, we predict electron pulse durations by weighting the different trajectories with distributions of the initial kinetic energy and emission angle and position, using procedures outlined in Refs. 21 and 27. The initial energy distribution is taken as the positive-energy half of a Gaussian centered at an energy of 0 eV, with a standard deviation (of the corresponding full Gaussian) of $\sigma_{E}$. For different initial kinetic energy widths, Fig. 2(d) displays the resulting electron pulse duration (full-width-at-half maximum, FWHM) in the energy range of 40–100 eV, derived from the distribution of arrival times at a plane 400 *μ*m behind the exit aperture of the gun. In particular, pulse widths below 1 ps are predicted throughout the energy range shown for initial energy widths of $\sigma_{E}\leq 0.5$ eV and at energies >70  eV for $\sigma_{E}\leq 1$ eV. Such energy widths were previously observed for two-photon photoemission from tungsten nanotips in Ultrafast Transmission Electron Microscopy (UTEM).^10^ The set of curves in Fig. 2(d) illustrates that both electron velocity dispersion and path length differences contribute to the final electron pulse duration. For the present design, path length differences amount to a pulse spreading of about 200–300 fs across the energy range plotted (red line, quasi-monochromatic initial energy distribution), which could be further reduced by the application of a higher extraction field or a smaller exit aperture.

## EXPERIMENTAL RESULTS

In the following, we experimentally characterize the pulse duration and the beam quality of the ultrafast photoelectron microgun. To this end, the gun is mounted inside an ultrahigh-vacuum (UHV) chamber (base pressure $7\times 10^{-10}$ mbar) and connected to computer-controlled voltage supplies. Two-photon photoelectron emission (identified by a quadratic intensity scaling of the photoemission current) is induced by focusing 400-nm femtosecond laser pulses (duration 80 fs, repetition rate 312 kHz, and pulse energy 110 pJ) onto the tungsten nanotip emitter using a plano-convex lens on a motorized linear 3D stage (focal length 2.3 mm focus diameter approx. 5 *μ*m), resulting in the emission of about one electron per pulse.^11,27,29^

**FIG. 3.**
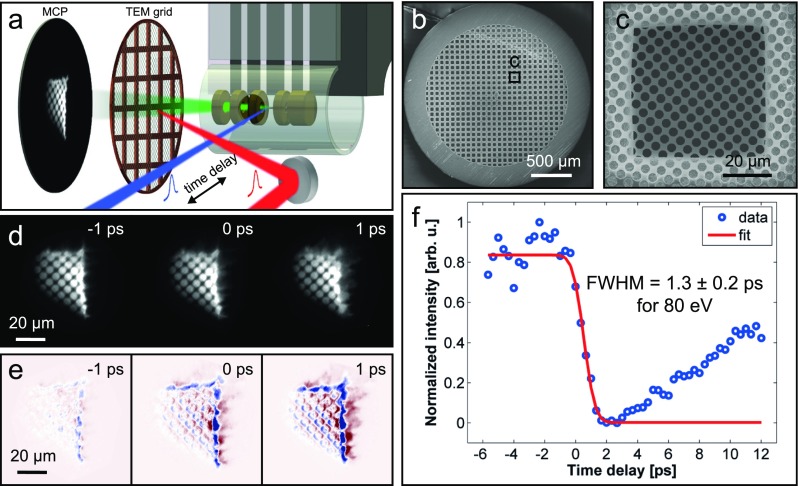
(a) Schematic of the experimental setup for characterizing electron-pulse durations via the transient electric field effect (not to scale). (b) and (c) TEM copper grid (square opening with 50 *μ*m width), covered with a perforated carbon film (Quantifoil, circular apertures of 3.5 *μ*m diameter). (d) Projection images recorded with photoelectrons from the microgun for different pump-probe time delays. (e) Difference in images taken at the given delay and a large negative delay (<−20) ps. (f) Contrast change fitted with an error function (red line), resulting in a temporal resolution of 1.3 ps or shorter at an electron energy of 80 eV.

The electron pulse duration is measured by using a previously established method based on transient electric fields.^29,33,34^ Specifically, the photoelectron beam is directed through a transmission electron microscope (TEM) copper mesh covered with a finer amorphous carbon grid (Figs. 3(a)–3(c)). For the lens potential at ground, a divergent beam is produced that results in a projection image of the TEM grid (Figs. 3(a) and 3(d)) with a magnification of about 200, which is recorded using a phosphor-screen microchannel plate (5 cm behind sample) and a CCD camera. In the pulse duration measurement, the projection image is distorted by a space-charge cloud near the sample, induced by an intense pump laser pulse (duration 80 fs, center wavelength 800 nm, and fluence up to 2 mJ/cm^2^), and the images are taken for variable optical-pump/electron-probe delays (see Figs. 3(a) and 3(d)). A delay-dependent series of projections (Fig. 3(d)) and difference images with respect to a fixed negative time delay (Fig. 3(e)) show a pump-induced contrast change over time. Evaluating the delay-dependent image contrast, we observe dynamical features as rapid as 1.3 ps (Fig. 3(f)), which represents an upper bound to the local electron pulse duration.

**FIG. 4.**
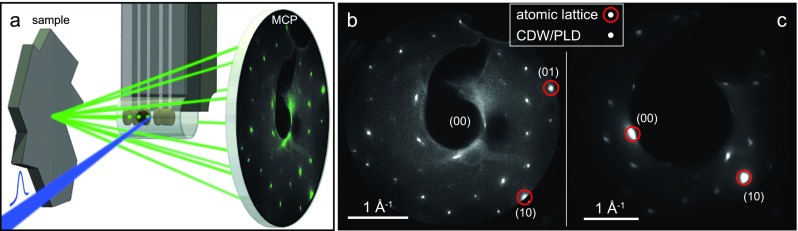
(a) Schematic of backscattering diffraction using the microgun (not to scale). Sample-detector distance: 55 mm. The maximum diffraction angle is approximately 35°. (b)–(c) LEED patterns of 1 T-TaS_2_ for an electron energy of 50 eV, corrected for distortions caused by the planar detector. Gun-sample distance: 550 *μ*m (b) and 270 *μ*m (c). Integration time: 100 s (b) and 600 s (c).

In Fig. 4, we demonstrate the operation of the microgun in backscattering diffraction-mode. LEED images from a single-crystalline surface of the transition metal dichalcogenide 1 T-TaS_2_ (cleaved in UHV) are recorded, as shown in Fig. 4(b). This material exhibits a periodic lattice distortion (PLD) associated with a charge density wave (CDW),^35^ which results in a complex pattern of superstructure diffraction spots. Atomic lattice Bragg spots (Fig. 4(b), red circles) and PLD spots of different orders (all other peaks) are clearly resolved. For approximate diffraction probabilities of 1%–3% and one incident electron per pulse, 10^5^–10^6^ electrons are detected in images within one to few minutes of exposure (Figs. 4(b) and 4(c)). In order to map a large number of Bragg conditions, the backscattered electron diffraction pattern was recorded with a gun-sample distance of 550 *μ*m. This gun-sample distance was determined using a series of diffraction patterns at different positions in front of the sample, using the linear scaling of the respective shadow diameters with the change in the working distance. Restricting the pattern to smaller parts of reciprocal space allows us to reduce the sample-gun distance to 270 *μ*m (Fig. 4(c)) or below, so that electron pulse durations as in the projection geometry (Fig. 3) are expected. From the minimum peak width observed (0.025 Å^−1^), we determine a transfer width of 25 nm. Combined with a beam diameter on the sample of approximately 3 *μ*m, we estimate a normalized beam emittance of 200 nm mrad. This emittance is compares favorably with most commercial LEED instruments and is largely caused by the small electron beam source size. A further reduction of the emittance may be achieved by reducing the exit aperture diameter.

## CONCLUSION

We described the fabrication and characterization of a micrometer-scale ultrafast photoelectron gun using photolithography and focused-ion-beam processing. Electron pulse widths of to 1.3 ps at 80 eV were observed, in agreement with numerical simulations for this gun geometry. Even shorter pulse durations could be achieved by further reducing the gun-sample distance, higher extraction fields at the tip apex, or by minimizing the photoemission excess energy using a lower photon energy. At present, the overall size of the gun assembly is limited by the breakdown voltages of the metallic lines and the thickness of the supporting substrate. Besides its benefits in the temporal resolution, the nanolocalized photoelectron source employed has demonstrated its potential to yield high-resolution LEED images. In the future, this microgun and its further developments will promote ultrafast LEED studies with picosecond and femtosecond temporal resolutions, providing direct access to structural dynamics at surfaces and surface reconstructions or in molecular adsorbate layers.
